# Associations between smoking, sex steroid hormones, trouble sleeping, and depression among U.S. adults: a cross-sectional study from NHANES (2013–2016)

**DOI:** 10.1186/s12889-024-19045-0

**Published:** 2024-06-07

**Authors:** Jing Huang, Peiwen Shi, Yuan Zhao, Huinan Zhang, Tian Gao, Xing Wang

**Affiliations:** Department of Health Management, Tangdu Hospital, Air Force Military Medical University, Xi’an, 710038 Shaanxi China

**Keywords:** Smoking, Sex steroid hormones, Trouble sleeping, Depression, NHANES

## Abstract

**Background:**

Dose-response and nonlinear relationships of cigarette exposure with sleep disturbances and depression are warranted, and the potential mechanism of sex hormones in such associations remains unclear.

**Methods:**

Cigarette exposure, trouble sleeping, and depression were assessed by standard questionnaires, and the levels of cotinine and sex steroid hormones were determined among 9900 adults from the National Health and Nutrition Examination Survey (NHANES). Multiple linear regression, logistic regression, and mediation models were conducted to evaluate the associations between smoking, sex steroid hormones, trouble sleeping, and depression.

**Results:**

With never smokers as a reference, current smokers had a higher prevalence of trouble sleeping (OR = 1.931, 95% *CI*: 1.680, 2.219) and depression (OR = 2.525, 95% *CI*: 1.936, 3.293) as well as testosterone level (β = 0.083, 95% *CI*: 0.028, 0.140). Pack-years of smoking and cigarettes per day were positively associated with the prevalence of trouble sleeping and depression as well as testosterone level (*P*_trend_ <0.05). The restricted cubic spline model showed linear relationships of cotinine with trouble sleeping, depression, and testosterone. The positive associations of cigarettes per day with trouble sleeping and depression were greater in females than that in males (*P*_modification_ <0.05). However, the potential role of sex hormones was not observed in the association of cotinine with trouble sleeping or depression (*P*_mediation_ >0.05).

**Conclusion:**

Smoking may induce sex hormone disturbance and increase the risk of sleep problems and depression symptoms, and ceasing smoking may reduce the risk of such complications.

**Supplementary Information:**

The online version contains supplementary material available at 10.1186/s12889-024-19045-0.

## Introduction

Depression has become a serious public health challenge worldwide, ranking among the leading causes of disability and poor quality of life [1–3]. The estimated prevalence of major depressive disorder is 5.5–14.6% from 18 high and low- to middle-income countries [4]. The number of incident cases of depression worldwide increased 49.9% from 1990 to 2017, and is still rising sharply [5]. Sleep disorders, including excessive or inadequate sleep and trouble sleeping, have been identified to be prodromes and precursors of depression [6]. Numerous cross-sectional and prospective studies have reported the associations of sleep duration and trouble sleeping with depression, suggesting that early screening and treatment of sleep disorders may reduce depression [7–9]. Identifying risk factors and further improving lifestyle to prevent trouble sleeping and depression is an urgent topic in the field of public health.

Smoking is one of the major risk factors for chronic noncommunicable diseases according to World Health Statistics 2023 by World Health Organization. A longitudinal study based on 7960 US adolescents showed a positive association between smoking status and the development of sleep problems [10]. Furthermore, a cross-sectional study conducted among a sample of 30,524 US adults from the National Health and Nutrition Examination Survey (NHANES) revealed that individuals who currently or previously smoked have a heightened risk of experiencing depression compared to those who have never smoked [11]. Although previous research suggests that smoking may contribute to trouble sleeping and depression, the majority of studies have primarily examined smoking status rather than the quantity of cigarettes consumed. Therefore, further exploration of the dose-response and nonlinear relationships between cigarette exposure and trouble sleeping as well as depression is needed. Cotinine, a major product of nicotine primary metabolism, is a widely used biomarker for the assessment of cigarette exposure, including active and passive smoking [12]. In addition, the underlying mechanisms for smoking-associated trouble sleeping and depression remain largely unclear.

Sex steroid hormones, including testosterone (TST), estrogens (EST) and sex hormone-binding globulin (SHBG), play a vital role in the physiology of reproductive tissue, bone, cardiovascular, lipid, and immune system [13]. TST and EST are involved in the promotion of secondary sexual characteristics in males and females and SHBG is a blood transport protein of TST and EST. Disruption of sex steroid hormones has been found to be associated with multiple adverse health effects, including sleep problems and depression [14, 15]. Meanwhile, previous studies have shown that smokers have significantly higher levels of TST and EST than non-smokers [16–19], indicating that cigarette exposure may disrupt sex steroid hormone levels. Therefore, we hypothesized that sex steroid hormones might play a potential role in the effects of trouble sleeping and depression induced by cigarette exposure.

In this study, the cross-sectional analyses were conducted among American adults from the 2013–2016 survey cycles of NHANES. Smoking status, trouble sleeping, and depression assessment were collected by questionnaires. Levels of cotinine and sex steroid hormones were tested using biological samples. Our objectives were to quantify the associations between cigarette exposure, sex steroid hormones (TST, EST and SHBG), trouble sleeping, and depression.

## Materials and methods

### Study population

The NHANES is a representative cross-sectional survey, which has been described elsewhere [20, 21]. Briefly, about 5,000 US civilians per year were recruited using a complex, multistage, probability design, conducted by Centers for Disease Control and Prevention (CDC)’s National Center for Health Statistics (NCHS), aiming to assess the health and nutritional status of adults and children in the United States. These persons are located in counties across the country, 15 of which are visited each year. Questionnaire interviews, physical examinations, and laboratory tests have been conducted every two years since 1999 to now, to obtain data on demographic, socioeconomic, dietary, physiological measurements, and biomarkers of health and environmental pollutants. The study protocol was approved by the NCHS Research Ethics Review Board, and all participants signed informed consent forms. The current analysis included 9900 adults over the age of 18 from the 2013 to 2016 NHANES cycles, the only two cycles in which sex steroid hormones were quantified. All the data in the present study was analyzed from January to June 2023 and is available on the NHANES website [22].

### Assessment of cigarette exposure

Cigarette exposure, including age started smoking, whether smoking now, time since quitting smoking, cigarettes per day during the past 30 days, and cigarettes per day when smoking cessation, were collected using standard questionnaires. Smoking status was classified as never smoker, former smoker, and current smoker. Pack-years of smoking were calculated by multiplying the cigarettes per day (pack/day) by the years of smoking for former and current smokers, and expressed as pack-year.

The concentrations of cotinine in serum samples were measured by an isotope-dilution high-performance liquid chromatography/atmospheric pressure chemical ionization tandem mass spectrometric (ID HPLC-APIC MS/MS) method. All quality control procedures recommended by the manufacturers were followed. Reported results for all assays meet the Division of Laboratory Sciences quality control and quality assurance performance criteria for accuracy and precision. The lower limit of detection (LLOD) for serum cotinine is 0.015 ng/mL. The values of serum cotinine below LLOD were replaced with LLOD/√2.

### Assessment of sex steroid hormones

The concentrations of serum total TST and EST were measured using isotope dilution liquid chromatography tandem mass spectrometry (ID-LC-MS/MS) method which was created for high sample throughput and demonstrates high accuracy and precision. Serum SHBG is based on the reaction of SHBG with immune-antibodies and chemo-luminescence measurements of the reaction products that occurs after two incubation periods and subjected to a magnetic field. The LLODs for TST, EST, and SHBG are 0.750 ng/mL, 2.994 pg/mL, and 0.800 nmol/L, respectively. The values of sex steroid hormones below LLOD were replaced with LLOD/√2.

### Assessment of trouble sleeping and depression

Trouble sleeping was defined by a questionnaire item “Have you ever told a doctor or other health professional that you have trouble sleeping”. Depression measurements were conducted by the Patient Health Questionnaire, in which a nine-item depression screening instrument was administered to determine the depression symptoms over the past 2 weeks. Every question was given a point range from 0 to 3 as the frequency of symptoms increased, and a score ≥ 10 indicated the presence of depressive symptoms [11].

### Covariates

Demographic and lifestyle data, including age, gender, body mass index (BMI), race, educational qualification, physical activity, alcohol consumption, ratio of family income to poverty, and regular periods (for females), were collected using standard questionnaires. Physical activity was divided into high-intensity, moderate-intensity, and no physical activity. High-intensity physical activity was defined as at least 10 min of continuous vigorous work or exercise per week causing large increases in breathing or heart rate. Moderate-intensity physical activity was defined as at least 10 min of continuous moderate more than vigorous work or exercise per week causing small increases in breathing or heart rate. Alcohol consumption was divided into yes (drinking at least once over the past 12 months) and no. Ratio of family income to poverty is a continuous variable ranging from 0 to 5.

### Statistical analysis

Missing values for BMI, educational qualification, ratio of family income to poverty, trouble sleeping, and cotinine were imputed with the use of the multiple imputation approach. The levels of sex steroid hormones and cotinine were natural logarithmically transformed owning to their right skewed distribution. Analysis of Variance, Kruskal-Wallis test, and chi-square test were conducted to compare the differences in basic characteristics between males and females for symmetrically distributed, right-skewed distributed, and categorical variables, respectively. Spearman’s rank correlation coefficient (*ρ*) was used to evaluate the correlation between sex steroid hormones: very weak (*ρ* < 0.20), weak (0.20 ≤ *ρ* < 0.40), moderate (0.40 ≤ *ρ* < 0.70), strong (0.70 ≤ *ρ* < 0.90), and very strong (*ρ* ≥ 0.90) [23]. The multiple linear regression model was used to estimate the associations between cigarette exposure and sex steroid hormones. The logistic regression model was used to estimate the associations between cigarette exposure and the prevalence of sleeping trouble and depression. Additionally, the restricted cubic spline (RCS) model was conducted to examine the potential linear/nonlinear relationship of serum cotinine with sex steroid hormone levels and the prevalence of sleeping trouble and depression. Given the gender differences in cigarette exposure, sex steroid hormone levels, and the prevalence of sleeping trouble and depression, stratified analyses were conducted by gender, and values of *P*_modification_ were estimated by adding a product term of gender and cigarette exposure to the regression model. The mediating roles of sex steroid hormone in the associations between serum cotinine and the prevalence of sleeping trouble and depression were investigated using mediation analyses in R software by the “mediation” package [24, 25]. Covariates were adjusted for age, gender, BMI, race, educational qualification, physical activity, alcohol consumption, ratio of family income to poverty, regular periods, and batch (survey cycle) in all models.

All *P* values were two sided with a statistically significant level at 0.05, and survey-weighted multiple linear and logistic regression analyses were performed with the “survey” package by R software.

## Results

### Basic characteristics

The basic characteristics of 9900 participants are presented in Table 1. The mean age of all subjects was 42.68 (standard deviation (SD) 15.11) years, with 4728 (47.76%) male. The proportions for never smokers, former smokers, and current smokers were 59.89%, 18.79%, and 21.32%, respectively. Trouble sleeping and depression rates were 25.49% and 8.17%, respectively. Females seemed to have higher levels of BMI, EST, and SHBG, as well as prevalence of trouble sleeping and depression, but lower levels of cotinine and TST than males (*P* < 0.05).

**Table 1:** Characteristics of survey participants

Characteristics	Total	Male	Female	*P*
N	9900	4728 (47.76)	5172 (52.24)	
Age, years	42.67 ± 15.11	42.72 ± 15.23	42.63 ± 14.99	0.759
BMI, km/m^2^	29.25 ± 7.33	28.68 ± 6.43	29.77 ± 8.04	< 0.001
Race				0.038
Mexican American	1662 (16.79)	783 (16.56)	879 (17.00)	
Other Hispanic	1139 (11.51)	500 (10.58)	639 (12.35)	
Non-Hispanic white	3336 (33.70)	1628 (34.43)	1708 (33.02)	
Non-Hispanic black	2165 (21.87)	1029 (21.76)	1136 (21.96)	
Other races	1598 (16.14)	788 (16.67)	810 (15.66)	
Educational qualification				< 0.001
Less than 9th grade	845 (8.54)	416 (8.80)	429 (8.29)	
9-11th grade	1262 (12.75)	662 (14.00)	600 (11.60)	
High school or equivalent	2173 (21.95)	1108 (23.43)	1065 (20.59)	
Some college or AA degree	3126 (31.58)	1355 (28.66)	1771 (34.24)	
College graduate or above	2494 (25.19)	1187 (25.11)	1307 (25.27)	
Ratio of family income to poverty	2.43 ± 1.64	2.51 ± 1.65	2.35 ± 1.63	< 0.001
Regular periods, yes	-	-	1783 (34.47)	-
Alcohol consumption, yes	6180 (62.42)	3232 (68.36)	2948 (57.00)	< 0.001
Physical activity				< 0.001
No physical activity	2906 (29.35)	1116 (23.60)	1790 (34.61)	
Moderate-intensity	2865 (28.94)	1125 (23.79)	1740 (33.64)	
High-intensity	4129 (41.71)	2487 (52.60)	1642 (31.75)	
Smoking status				< 0.001
Never smoker	5929 (59.89)	2422 (51.23)	3507 (67.81)	
Former smoker	1860 (18.79)	1117 (23.63)	743 (14.37)	
Current smoker	2111 (21.32)	1189 (25.15)	922 (19.18)	
Serum cotinine, ng/mL	0.04 (0.01, 22.50)	0.07 (0.01, 90.88)	0.03 (0.01, 0.86)	< 0.001
Sex steroid hormones				
TST, ng/L	570.50 (199.00, 3920.00)	4000.00 (3020.00, 5240.00)	206.00 (141.00, 295.00)	< 0.001
EST, ng/L	24.80 (15.20, 42.30)	23.80 (18.60, 29.60)	31.80 (7.23, 96.23)	< 0.001
SHBG, nmol/L	45.09 (30.52, 69.05)	35.51 (25.51, 49.89)	57.64 (38.53, 88.96)	< 0.001
Trouble sleeping, yes	2524 (25.49)	1026 (21.70)	1498 (28.96)	< 0.001
Depression, yes	809 (8.17)	292 (6.18)	517 (10.00)	< 0.001

Definition of abbreviations: BMI = body mass index; TST = testosterone; EST = estrogens; SHBG = sex hormone-binding globulin. Data are presented as mean (standard deviation), median (quartile range), and number (percentage) for symmetric distributed, skewed distributed, and categorical variables, respectively

The Spearman’s rank correlation coefficients between sex steroid hormones are shown in figure S1. Among sex steroid hormones, very weak positive correlations were presented between TST and EST, and EST and SHBG, whereas a very weak negative correlation was presented between TST and SHBG.

### Associations of cigarette exposure with trouble sleeping and depression

As shown in Tables 2 and 3, smoking status was positively associated with the prevalence of trouble sleeping and depression. With never smokers as a reference, the prevalence of trouble sleeping increased by 57.8% (95% *CI*: 1.368, 1.821) and 93.1% (95% *CI*: 1.680, 2.219), and the prevalence of depression increased by 33.4% (95% *CI*: 1.003, 1.773) and 152.5% (95% *CI*: 1.936, 3.293) in former and current smokers, respectively. Meanwhile, the prevalence of trouble sleeping and depression in current smokers significantly increased when former smokers as a reference (*P* < 0.05). In addition, the prevalence of trouble sleeping and depression were significantly increased with the elevated quartiles of pack-years of smoking and cigarettes per day (*P* and *P*_trend_ <0.05). In subgroup analyses among males and females, the significantly positive associations between cigarette exposure and the prevalence of trouble sleeping and depression did not change appreciably. Notably, the associations of cigarettes per day with the prevalence of trouble sleeping and depression were greater in females than that in males (*P*_modification_ <0.05). Additionally, linear relationships between the cotinine level and the prevalence of trouble sleeping and depression were shown using the RCS model (figure S2, *P* for total < 0.05 and *P* for nonlinear > 0.05), while the association between cotinine and depression prevalence appeared to be J-shaped in females (*P* for total and *P* for nonlinear < 0.05).

**Table 2:** Association of cigarette exposure with trouble sleeping

Cigarette exposure	OR (95% CI)			
	Total	Male	Female	*P*_modification_
Smoking status				0.668
Never	1 (ref.)	1 (ref.)	1 (ref.)	
Former	1.578 (1.368, 1.821) ^***^	1.734 (1.335, 2.253) ^***^	1.453 (1.188, 1.777) ^**^	
Current	1.931 (1.680, 2.219) ^***§^	1.944 (1.577, 2.398) ^***^	1.978 (1.592, 2.458) ^***§^	
Pack-years of smoking	1.007 (1.002, 1.012) ^**^	1.006 (1.002, 1.010) ^*^	1.009 (1.003, 1.016) ^*^	0.363
Never-smokers	1 (ref.)	1 (ref.)	1 (ref.)	
Q1	1.524 (1.246, 1.865) ^**^	1.576 (1.216, 2.042) ^**^	1.521 (1.058, 2.189) ^*^	
Q2	1.730 (1.350, 2.217) ^***^	1.927 (1.415, 2.625) ^***^	1.651 (1.165, 2.338) ^**^	
Q3	1.811 (1.457, 2.253) ^***^	1.775 (1.348, 2.336) ^***^	1.891 (1.243, 2.876) ^**^	
Q4	1.859 (1.468, 2.353) ^***^	1.973 (1.502, 2.593) ^***^	1.675 (1.256, 2.233) ^**^	
*P*_trend_	< 0.001	< 0.001	< 0.001	
Cigarettes per day now	1.032 (1.023, 1.042) ^***^	1.024 (1.009, 1.039) ^**^	1.046 (1.033, 1.060) ^***^	0.049
Non-smokers	1 (ref.)	1 (ref.)	1 (ref.)	
Q1	1.182 (0.901, 1.552)	1.136 (0.645, 1.999)	1.224 (0.769, 2.013)	
Q2	1.581 (1.094, 2.286) ^*^	1.628 (1.087, 2.439) ^*^	1.425 (0.880, 2.308)	
Q3	1.958 (1.533, 2.499) ^***^	1.731 (1.177, 2.546) ^*^	2.026 (1.344, 3.053) ^**^	
Q4	1.787 (1.404, 2.276) ^***^	1.548 (1.036, 2.315) ^*^	2.201 (1.635, 2.963) ^***^	
*P*_trend_	< 0.001	0.001	< 0.001	

Models were adjusted for age, gender, BMI, race, educational qualification, physical activity, alcohol consumption, ratio of family income to poverty, regular periods, and batch (survey cycle). *P*_trend_ was tested by including never-/non-smokers, Q1, Q2, Q3, and Q4 as 1, 2, 3, 4, 5 (continuous variable) in models, respectively. ^*^P < 0.05, ^**^P < 0.01, ^***^P < 0.001. ^§^former smokers were set as a reference. ^§^*P* < 0.05, ^§§^*P* < 0.01, ^§§§^*P* < 0.001

**Table 3:** Association of cigarette exposure with depression

Cigarette exposure	OR (95% CI)			
	Total	Male	Female	*P*_modification_
Smoking status				0.668
Never	1 (ref.)	1 (ref.)	1 (ref.)	
Former	1.334 (1.003, 1.773) ^*^	1.364 (0.799, 2.328)	1.281 (0.804, 2.043)	
Current	2.525 (1.936, 3.293) ^***§§§^	2.258 (1.424, 3.582) ^**§^	2.660 (1.946, 3.637) ^***§§§^	
Pack-years of smoking	1.007 (1.002, 1.012) ^*^	1.004 (0.997, 1.011)	1.012 (1.004, 1.020) ^**^	0.154
Never-smokers	1 (ref.)	1 (ref.)	1 (ref.)	
Q1	1.755 (1.121, 2.747) ^*^	2.029 (1.112, 3.701) ^*^	1.586 (0.974, 2.582)	
Q2	1.530 (1.091, 2.144) ^*^	1.532 (0.738, 3.181)	1.491 (0.913, 2.435)	
Q3	2.062 (1.466, 2.902) ^***^	1.740 (1.027, 2.949) ^*^	2.051 (1.256, 3.349) ^**^	
Q4	2.424 (1.624, 3.615) ^***^	1.793 (1.124, 2.862) ^*^	2.675 (1.556, 4.599) ^**^	
*P*_trend_	< 0.001	0.011	< 0.001	
Cigarettes per day now	1.044 (1.029, 1.058) ^***^	1.021 (0.998, 1.044)	1.067 (1.044, 1.092) ^***^	0.011
Non-smokers	1 (ref.)	1 (ref.)	1 (ref.)	
Q1	1.798 (1.054, 3.068) ^*^	1.908 (0.835, 4.356)	1.567 (0.870, 2.824)	
Q2	1.869 (1.188, 2.940) ^*^	2.321 (1.366, 3.945) ^**^	1.633 (1.040, 2.562) ^*^	
Q3	2.032 (1.384, 2.981) ^**^	2.373 (1.189, 4.736) ^*^	2.027 (1.339, 3.069) ^**^	
Q4	3.025 (2.188, 4.181) ^***^	1.494 (0.807, 2.766)	4.020 (2.603, 6.207) ^***^	
*P*_trend_	< 0.001	0.008	< 0.001	

Models were adjusted for age, gender, BMI, race, educational qualification, physical activity, alcohol consumption, ratio of family income to poverty, regular periods, and batch (survey cycle). *P*_trend_ was tested by including never-/non-smokers, Q1, Q2, Q3, and Q4 as 1, 2, 3, 4, 5 (continuous variable) in models, respectively. ^*^P < 0.05, ^**^P < 0.01, ^***^P < 0.001. ^§^former smokers were as a reference. ^§^*P* < 0.05, ^§§^*P* < 0.01, ^§§§^*P* < 0.001

### Associations of cigarette exposure with sex steroid hormones

As presented in Table 4, with never smokers as a reference, the level of TST increased by 8.3% (95% *CI*: 2.8%, 14.0%) in current smokers, whereas the difference with former smokers was insignificant. When former smokers as a reference, the level of TST significantly increased, but EST significantly decreased in current smokers (*P* < 0.05). Each 1 cigarette per day increase in current smokers was associated with increased levels of TST and SHBG for 0.5% (0.2%, 0.7%) and 0.3% (0.1%, 0.6%), respectively. Meanwhile, the estimated changes of TST and SHBG were significantly increased with the elevated quartiles of cigarettes per day (*P*_trend_ <0.05). However, similar association was not observed between the levels of sex steroid hormones and pack-years of smoking (*P* and *P*_trend_ >0.05). In subgroup analyses (table S7), the associations of current smoking status and amount with the TST level remained significant (*P* and *P*_trend_ <0.05), while the associations of current smoking status and amounts with the SHBG level were significant in males rather than females. Furthermore, the RCS models also yielded a linear relationship between serum cotinine concentrations and TST and SHBG levels (figure S3, *P* for total < 0.05 and *P* for nonlinear > 0.05).

**Table 4:** Association of cigarette exposure with sex steroid hormone levels

Cigarette exposure	Estimated changes (95% CI)		
	TST	EST	SHBG
Smoking status			
Never	0 (ref.)	0 (ref.)	0 (ref.)
Former	0.013 (-0.038, 0.068)	0.037 (-0.040, 0.121)	0.000 (-0.039, 0.041)
Current	0.083 (0.028, 0.140) ^**§§^	-0.061 (-0.123, 0.006) ^§^	0.038 (-0.010, 0.088)
Pack-years of smoking	0.000 (-0.001, 0.001)	0.000 (-0.002, 0.002)	0.001 (0.000, 0.002)
Never-smokers	0 (ref.)	0 (ref.)	0 (ref.)
Q1	0.033 (-0.025, 0.095)	-0.053 (-0.147, 0.053)	-0.002 (-0.056, 0.054)
Q2	0.046 (-0.035, 0.133)	0.031 (-0.058, 0.129)	-0.012 (-0.055, 0.033)
Q3	0.062 (-0.030, 0.161)	-0.027 (-0.109, 0.062)	0.028 (-0.036, 0.096)
Q4	0.045 (-0.026, 0.121)	0.007 (-0.089, 0.113)	0.059 (-0.009, 0.133)
*P*_trend_	0.080	0.995	0.117
Cigarettes per day now	0.005 (0.002, 0.007) ^**^	-0.003 (-0.006, 0.001)	0.003 (0.001, 0.006) ^*^
Non-smokers	0 (ref.)	0 (ref.)	0 (ref.)
Q1	0.037 (-0.037, 0.116)	-0.150 (-0.258, -0.027) ^*^	0.021 (-0.046, 0.093)
Q2	0.090 (0.015, 0.171) ^*^	-0.034 (-0.121, 0.061)	-0.016 (-0.077, 0.048)
Q3	0.112 (0.019, 0.212) ^*^	-0.090 (-0.226, 0.071)	0.036 (-0.027, 0.104)
Q4	0.076 (0.013, 0.142) ^*^	-0.040 (-0.118, 0.045)	0.085 (0.024, 0.151) ^*^
*P*_trend_	0.003	0.069	0.018

Models were adjusted for age, gender, BMI, race, educational qualification, physical activity, alcohol consumption, ratio of family income to poverty, regular periods, and batch (survey cycle). *P*_trend_ was tested by including never-/non-smokers, Q1, Q2, Q3, and Q4 as 1, 2, 3, 4, 5 (continuous variable) in models, respectively. All values were calculated using exp^β^-1, due to the natural logarithmic transformation of sex steroid hormones. ^*^P < 0.05, ^**^P < 0.01, ^***^P < 0.001. ^§^former smokers were as a reference. ^§^*P* < 0.05, ^§§^*P* < 0.01, ^§§§^*P* < 0.001

### Associations of sex steroid hormones with trouble sleeping and depression

**Fig. 1:**
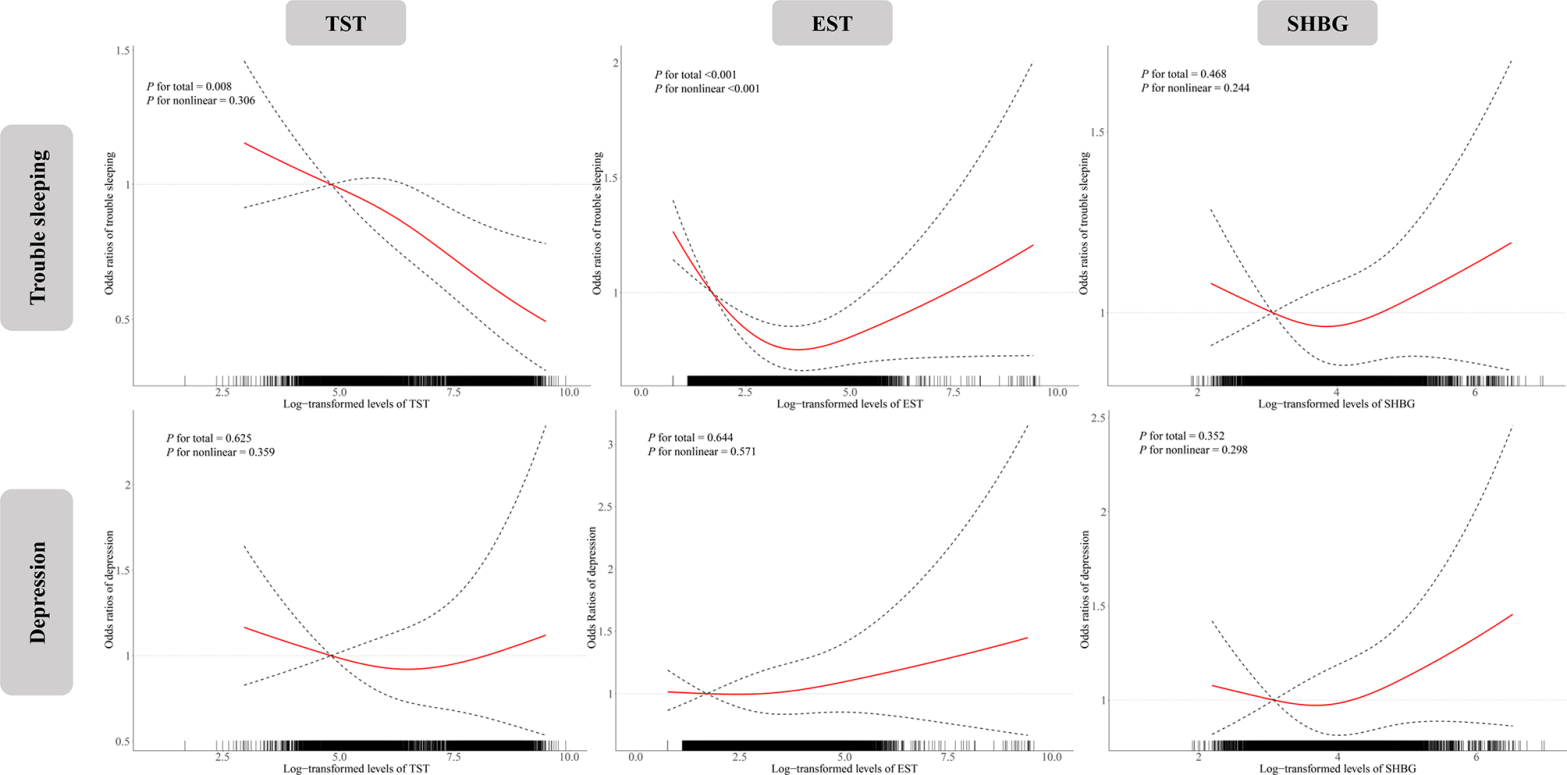
**Association of sex steroid hormones with trouble sleeping and depression.** Models were adjusted for age, gender, BMI, race, educational qualification, physical activity, alcohol consumption, ratio of family income to poverty, regular periods, and batch (survey cycle). Knots were placed at the 5th, 50th, and 95th percentiles of sex steroid hormone distributions, and the reference value was set at the 10th percentile

The associations between sex steroid hormones and the prevalence of trouble sleeping and depression are presented in Fig. 1. The RCS models showed a linear negative relationship between TST and trouble sleeping prevalence (*P* for total < 0.05 and *P* for nonlinear > 0.05) and a U-shaped relationship between EST and trouble sleeping prevalence (*P* for total and *P* for nonlinear < 0.05). In addition, trouble sleeping was observed to present a U-shaped relationship with TST in males and with EST in females (figure S4, *P* for total and *P* for nonlinear < 0.05). Although the association between sex steroid hormones and depression prevalence was not observed in total participants (*P* for total and *P* for nonlinear > 0.05), U-shaped relationships of TST and SHBG levels with depression prevalence were shown among males in subgroup analyses (figure S5, *P* for total and *P* for nonlinear < 0.05).

### Roles of sex steroid hormones in the associations between cigarette exposure and the prevalence of trouble sleeping and depression

The mediating effects of sex steroid hormones in the associations between serum cotinine and the prevalence of trouble sleeping and depression are presented in Table 5. Indeed, the mediating effect of TST, EST, or SHBG was not observed in the association between cotinine concentrations and trouble sleeping or depression (*P*_mediating_ >0.05).

**Table 5:** Mediating effects of sex steroid hormone on the associations of serum cotinine with trouble sleeping and depression

Outcome/sex steroid hormone	Total effects of cotinine	Direct effects of cotinine	Proportion mediated by sex steroid hormone	*P*_mediation_
Trouble sleeping				
TST	1.058 (1.045, 1.072)	1.060 (1.046, 1.074)	NA	NA
EST		1.058 (1.045, 1.072)	0.18%	0.492
SHBG		1.058 (1.045, 1.072)	0.10%	0.830
Depression				
TST	1.097 (1.075, 1.118)	1.097 (1.076, 1.119)	NA	NA
EST		1.097 (1.076, 1.119)	NA	NA
SHBG		1.096 (1.075, 1.118)	0.40%	0.422

Total effects were adjusted for age, gender, BMI, race, educational qualification, physical activity, alcohol consumption, ratio of family income to poverty, regular periods, and batch (survey cycle). Direct effects additionally adjusted sex steroid hormone. NA represents that it is not applicable to calculate the mediation ratio, since the direct effect is greater than the total effect

## Discussion

In this study, we found that cigarette exposure, both former and current smoking, was positively associated with the prevalence of trouble sleeping and depression, and the associations of current cigarette consumption with trouble sleeping and depression were stronger in females than that in males. Current rather than former or total cigarette exposure was positively associated with the levels of TST and SHBG, whereas trouble sleeping prevalence showed a negative linear association with TST and a U-shaped association with EST. However, the potential role of sex steroid hormones was not observed in the association of cigarette exposure with the prevalence of trouble sleeping or depression.

The effects of smoking on trouble sleeping and depression have been reported in epidemiological studies. In studies on the effects of smoking on sleep, A cross-sectional analysis from 3233 subjects demonstrated that current smokers had decreased objective sleep quality than former or never smokers, with a dose-dependent association between smoking intensity and indicators of polysomnography [26]. A clinical study based on 32 participants found that poorer sleep quality was positively associated with increased craving and smoking urges, and suggested that poor sleep quality in cigarette smokers is associated with greater nicotine dependence [27]. A cross-sectional study among 30,097 Canadian community reported higher prevalence of insomnia and shorter sleep duration in daily smokers [28]. Similar associations have been reported among non-Hispanic adolescents [29]. Regarding depression, a cohort study involving 924 students found that early adolescent smoking predicted subsequent depression [30]. Another study with a sample size of 248,800 US adults revealed that the prevalence of depression was higher among current smokers compared to former smokers. Additionally, individuals who attempted to quit smoking but were unsuccessful (unsuccessful quitters) experienced higher levels of depression compared to both former smokers and non-quitters (individuals who made no attempts to quit) [31]. In line with these previous studies, our own research demonstrated that both current and former smoking were associated with an increased risk of trouble sleeping and depression, with the risk becoming more pronounced with higher smoking intensity. Notably, we found that the association between smoking intensity and trouble sleeping as well as depression was greater in females than in males. Females have nearly twice the prevalence of depression than males, and gender differences in depression due to gender inequality, economic problems, psychological changes caused by childbirth, social pressures, hormonal differences, and neurodevelopmental changes make females more vulnerable to smoking-induced depression [32]. These findings underscore the importance of giving more attention to psychological issues among females.

Studies have shown that smoking is one of the main contributing factors of sex steroid hormone disorders. A meta-analysis in 28 studies of 13,317 men and 6089 women reported that smokers had a higher level of TST than non-smokers [33]. Results from 65,000 postmenopausal women presented that current smokers had higher levels of TST than non-smokers, and there was a dose-response relationship between smoking amount and TST levels [34]. Another cross-sectional study among 2030 postmenopausal women also showed that the daily number of cigarettes smoked was positively associated with the levels of TST, EST, and SHBG among current smokers [35]. Notably, this study demonstrated that sex steroid hormone levels in former smokers returned to the levels of never smokers within 1–2 years of smoking cessation, which aligns with our findings. In our study, the TST level was higher in current smokers than never smokers, and the number of cigarettes per day was positively associated with the TST and SHBG levels, whereas there was no significant difference in sex steroid hormone levels between former and never smokers. These findings suggest that smoking may only have a short-term effect on sex steroid hormone disruption, and smoking cessation may reduce the risk of hormone-related diseases.

Epidemiological studies showed that women experience more sleep problems and depressive symptoms around times when sex hormones change, such as puberty and menopause [32]. But the effect of sex hormones on sleep problems and depression remain unclear. Worse sleep was reported among depressed patients than controls, but the difference in the levels of endogenous hormones was insignificant [14]. Zambotti et al. conducted a cross-sectional laboratory study among 33 perimenopausal women, in which sleep problems were not associated with sex hormones, but instead appeared to be influenced by other factors including hyperactivity, poor mood, and night-to-night variability [36]. In a randomized double-blind placebo-controlled trial conducted by Dichtel et al., adjunctive transdermal TST was not more effective than placebo in improving symptoms of depression among women with antidepressant-resistant major depression in 8-week treatment [37]. In this study among general adults, trouble sleeping was negatively and U-shaped associated with TST and EST, respectively, but depression was not significantly associated with sex steroid hormones. More researches are necessary to confirm the relationship of sex hormones with sleep problems and depression.

The potential role of sex hormones was not observed in the association of cigarette exposure with trouble sleeping or depression in the present study. At present, the potential mechanisms of smoking causing sleep problems mainly include the following. First, several neurotransmitters, including acetylcholine, dopamine, serotonin, norepinephrine, and gamma-aminobutyric acid, are released caused by activating nicotinic receptors by cigarette exposure, and furtherly influence the central mechanisms that regulate the sleep-wake cycle [38]. Secondly, smokers may experience withdrawal symptoms and cravings when preparing to fall asleep or after falling asleep due to reduced nicotine level [39]. Additionally, Liu et al. found that Pittsburgh Sleep Quality Index scores and TNF-α levels in cerebrospinal fluid were higher in active smokers than that in non-smokers, revealing the potential role of neuroinflammation on the effect of poor sleep quality induced by smoking [40]. Further research is needed to explain the potential mechanisms of smoking on the adverse effects of trouble sleeping and depression.

The present study analyzed associations of smoking, sex steroid hormones, trouble sleeping, and depression among a relatively large population. Our founding provided new information of cigarette exposure on the adverse health effects. Nevertheless, there are some limitations in our study. First, the cross-sectional design limits inference on the temporality, and a casual effect needs to be confirmed in prospective cohort studies. Second, other covariates (dietary assessment, gene, environmental pollutants, etc.) that may affect sex steroid hormones, trouble sleeping, and depression were not considered. Third, cotinine and sex steroid hormones in biological samples are relatively unstable over long periods of time, and a trajectory model with repeated measurement is warranted.

## Conclusions

In the present study, we found that current smokers had higher levels of TST and a greater prevalence of trouble sleeping and depression compared to never smokers and former smokers. Serum cotinine level was linearly associated with TST level and the prevalence of trouble sleeping and depression. These findings indicate that never smoking or smoking cessation may prevent diseases related to sex hormone disorders and reduce the risk of sleep problems and depression symptoms. Interestingly, mediation analysis did not identify sex hormones as potential mediators for the association between cigarette exposure and the prevalence of trouble sleeping or depression.

### Electronic supplementary material

Below is the link to the electronic supplementary material.

Supplementary Material 1

## Data Availability

All the data in the present analysis is available at NHANES website https://www.cdc.gov/nchs/nhanes/index.htm.

## Abbreviations

NHANES: National Health and Nutrition Examination Survey
TST: testosterone
EST: estrogens
SHBG: sex hormone-binding globulin
CDC: Centers for Disease Control and Prevention
NCHS: National Center for Health Statistics
ID-HPLC-APCI-MS/MS: isotope-dilution high-performance liquid chromatography/atmospheric pressure chemical ionization tandem mass spectrometric
LLOD: lower limit of detection
ID-LC-MS/MS: isotope dilution liquid chromatography tandem mass spectrometry
BMI: body mass index
RCS: restricted cubic spline
SD: standard deviation
