# Supplementary material for: Associations between smoking, sex steroid hormones, trouble sleeping, and depression among U.S. adults: a cross-sectional study from NHANES (2013–2016)
Source: BMC Public Health. 2024 Jun 7;24:1541. doi: 10.1186/s12889-024-19045-0 (PMC11157951; doi:10.1186/s12889-024-19045-0)
Supplement: Supplementary file 1 — Supplementary Material 1 [file 12889_2024_19045_MOESM1_ESM.docx]

Table S1. Distribution of missing values

| Variable | N (%) |
| --- | --- |
| BMI | 111 (1.12%) |
| Educational qualification | 600 (6.06%) |
| Ratio of family income to poverty | 858 (8.67%) |
| Trouble sleeping | 1 (0.01%) |
| Cigarettes per day during past 30 days for current smokers | 29 (0.29%) |
| Cigarettes per day when quit for former smokers | 16 (0.16%) |
| Cotinine | 514 (5.19%) |


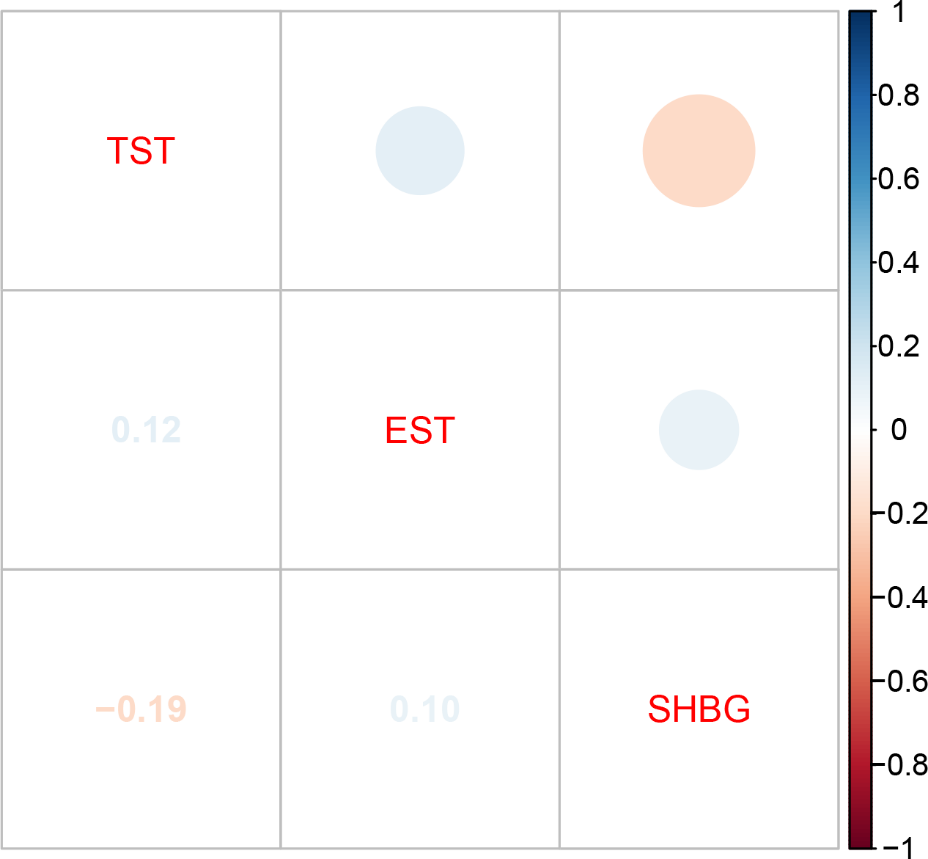


Figure S1. Spearman’s rank correlation coefficients between sex steroid hormones.

Table S2. Association of self-reported cigarette exposure with serum cotinine

| Self-reported cigarette exposure | Estimated changes (95% *CI*) of serum cotinine |
| --- | --- |
| Smoking status |  |
| Never | 0 (ref.) |
| Former | 0.441 (0.209, 0.718) ^***^ |
| Current | 1.120 (0.895, 1.372) ^***^ |
|  |  |
| Pack-years of smoking | 0.004 (0.001, 0.007) ^**^ |
| Never-smokers | 0 (ref.) |
| Q1 | 0.839 (0.623, 1.082) ^***^ |
| Q2 | 1.056 (0.807, 1.339) ^***^ |
| Q3 | 1.062 (0.755, 1.423) ^***^ |
| Q4 | 1.009 (0.679, 1.042) ^***^ |
| *P*_trend_ | <0.001 |
|  |  |
| Cigarettes per day now | 0.024 (0.020, 0.028) ^***^ |
| Non-smokers | 0 (ref.) |
| Q1 | 0.394 (0.248, 0.556) ^***^ |
| Q2 | 0.846 (0.723, 0.977) ^***^ |
| Q3 | 0.961 (0.836, 1.095) ^***^ |
| Q4 | 0.890 (0.754, 1.036) ^***^ |
| *P*_trend_ | <0.001 |

Models were adjusted for age, gender, BMI, race, educational qualification, physical activity, alcohol consumption, ratio of family income to poverty, regular periods, and batch (survey cycle). *P*_trend_ was tested by including never-/non-smokers, Q1, Q2, Q3, and Q4 as 1, 2, 3, 4, 5 (continuous variable) in models, respectively. All values were calculated using exp^β^-1, due to the natural logarithmic transformation of sex steroid hormones. ^*^*P*<0.05, ^**^*P*<0.01, ^***^*P*<0.001.

Table S3. Association of trouble sleeping with depression

| Trouble sleeping | Odds ratios of depression | *P* value |
| --- | --- | --- |
| Model 1 |  |  |
| No | 1 (ref.) |  |
| Yes | 5.680 (4.806, 6.713) | <0.001 |
| Model 2 |  |  |
| No | 1 (ref.) |  |
| Yes | 5.457 (4.544, 6.555) | <0.001 |
| Model 3 |  |  |
| No | 1 (ref.) |  |
| Yes | 5.427 (4.497, 6.549) | <0.001 |
| Model 4 |  |  |
| No | 1 (ref.) |  |
| Yes | 5.115 (4.246, 6.161) | <0.001 |

Model 1 was crude model.

Model 2 was adjusted for age, gender, BMI, and batch (survey cycle).

Model 3 was adjusted for race, educational qualification, physical activity, alcohol consumption, ratio of family income to poverty, and regular periods on the basis of model 2.

Model 4 was adjusted for cigarette smoking on the basis of model 3.

Table S4. Associations of cigarette exposure with trouble sleeping and depression (crude model)

| Cigarette exposure | OR (95% *CI*) | |
| --- | --- | --- |
|  | Trouble sleeping | Depression |
| Smoking status |  |  |
| Never | 1 (ref.) | 1 (ref.) |
| Former | 1.906 (1.656, 2.193) ^***^ | 1.467 (1.122, 1.920) ^**^ |
| Current | 1.949 (1.687, 2.251) ^***^ | 3.408 (2.655, 4.375) ^***^ |
|  |  |  |
| Pack-years of smoking | 1.013 (1.008, 1.018) ^*^^**^ | 1.009 (1.005, 1.013) ^***^ |
| Never-smokers | 1 (ref.) | 1 (ref.) |
| Q1 | 1.305 (1.098, 1.550) ^**^ | 1.994 (1.335, 2.979) ^***^ |
| Q2 | 1.728 (1.407, 2.122) ^***^ | 1.935 (1.431, 2.616) ^***^ |
| Q3 | 2.148 (1.756, 2.628) ^***^ | 2.634 (1.943, 3.571) ^***^ |
| Q4 | 2.565 (2.048, 3.214) ^***^ | 2.838 (1.992, 4.044) ^***^ |
| *P*_trend_ | <0.001 | <0.001 |
|  |  |  |
| Cigarettes per day now | 1.037 (1.028, 1.045) ^***^ | 1.059 (1.046, 1.072) ^***^ |
| Non-smokers | 1 (ref.) | 1 (ref.) |
| Q1 | 0.953 (0.750, 1.210) | 2.065 (1.269, 3.361) ^**^ |
| Q2 | 1.379 (1.012, 1.877) ^*^ | 2.434 (1.637, 3.619) ^***^ |
| Q3 | 2.035 (1.673, 2.477) ^***^ | 2.923 (2.051, 4.167) ^***^ |
| Q4 | 1.964 (1.570, 2.459) ^***^ | 4.109 (3.046, 5.544) ^***^ |
| *P*_trend_ | <0.001 | <0.001 |

Models were not adjusted for any covariate. *P*_trend_ was tested by including never-/non-smokers, Q1, Q2, Q3, and Q4 as 1, 2, 3, 4, 5 (continuous variable) in models, respectively. ^*^*P*<0.05, ^**^*P*<0.01, ^***^*P*<0.001.

Table S5. Association of cigarette exposure with sex steroid hormone levels (crude model)

| Cigarette exposure | Estimated changes (95% *CI*) | | |
| --- | --- | --- | --- |
|  | TST | EST | SHBG |
| Smoking status |  |  |  |
| Never | 0 (ref.) | 0 (ref.) | 0 (ref.) |
| Former | 0.409 (0.277, 0.554) ^***^ | -0.190 (-0.257, -0.117) ^***^ | -0.068 (-0.105, -0.030) ^**^ |
| Current | 0.407 (0.259, 0.573) ^***^ | -0.135 (-0.211, -0.051) ^**^ | -0.002 (-0.053, 0.051) |
|  |  |  |  |
| Pack-years of smoking | 0.006 (0.004, 0.008) ^***^ | -0.007 (-0.009, -0.005) ^***^ | 0.000 (-0.001, 0.001) |
| Never-smokers | 0 (ref.) | 0 (ref.) | 0 (ref.) |
| Q1 | 0.478 (0.260, 0.734) ^***^ | 0.038 (-0.067, 0.156) | -0.083 (-0.139, -0.024) ^**^ |
| Q2 | 0.307 (0.152, 0.483) ^***^ | 0.024 (-0.099, 0.163) | -0.055 (-0.100, -0.009) ^*^ |
| Q3 | 0.220 (0.074, 0.386) ^***^ | -0.233 (-0.312, -0.145) ^***^ | 0.015 (-0.050, 0.085) |
| Q4 | 0.618 (0.469, 0.782) ^***^ | -0.366 (-0.429, -0.297) ^***^ | -0.025 (-0.084, 0.037) |
| *P*_trend_ | <0.001 | <0.001 | 0.485 |
|  |  |  |  |
| Cigarettes per day now | 0.018 (0.011, 0.025) ^***^ | -0.009 (-0.014, -0.003) ^**^ | 0.002 (-0.001, 0.006) |
| Non-smokers | 0 (ref.) | 0 (ref.) | 0 (ref.) |
| Q1 | 0.550 (0.278, 0.880) ^***^ | -0.044 (-0.161, 0.090) | -0.082 (-0.147, -0.011) ^*^ |
| Q2 | 0.195 (0.020, 0.399) ^*^ | 0.046 (-0.0671, 0.173) | -0.025 (-0.091, 0.046) |
| Q3 | -0.069 (-0.219, 0.110) | -0.095 (-0.240, 0.077) | 0.120 (0.047, 0.199) ^**^ |
| Q4 | 0.505 (0.285, 0.764) ^***^ | -0.182 (-0.278, -0.074) ^**^ | 0.035 (-0.036, 0.110) ^*^ |
| *P*_trend_ | <0.001 | 0.007 | 0.132 |

Models were not adjusted for any covariate. *P*_trend_ was tested by including never-/non-smokers, Q1, Q2, Q3, and Q4 as 1, 2, 3, 4, 5 (continuous variable) in models, respectively. All values were calculated using exp^β^-1, due to the natural logarithmic transformation of sex steroid hormones.

Table S6. Association of cigarette exposure with depression (adjusted for trouble sleeping)

| Cigarette exposure | Odds ratios of depression |
| --- | --- |
| Smoking status |  |
| Never | 1 (ref.) |
| Former | 1.157 (0.850, 1.574) |
| Current | 2.097 (1.572, 2.798) ^***^ |
|  |  |
| Pack-years of smoking | 1.005 (1.000, 1.011) |
| Never-smokers | 1 (ref.) |
| Q1 | 1.557 (0.985, 2.461) |
| Q2 | 1.307 (0.912, 1.874) |
| Q3 | 1.686 (1.199, 2.371) ^**^ |
| Q4 | 1.977 (1.249, 3.129) ^**^ |
| *P*_trend_ | 0.001 |
|  |  |
| Cigarettes per day now | 1.034 (1.018, 1.051) ^***^ |
| Non-smokers | 1 (ref.) |
| Q1 | 1.742 (0.999, 3.038) |
| Q2 | 1.642 (1.081, 2.494) ^*^ |
| Q3 | 1.628 (1.097, 2.419) ^*^ |
| Q4 | 2.618 (1.795, 3.819) ^***^ |
| *P*_trend_ | <0.001 |

Models were adjusted for age, gender, BMI, race, educational qualification, physical activity, alcohol consumption, ratio of family income to poverty, regular periods, trouble sleeping, and batch (survey cycle). *P*_trend_ was tested by including never-/non-smokers, Q1, Q2, Q3, and Q4 as 1, 2, 3, 4, 5 (continuous variable) in models, respectively. ^*^*P*<0.05, ^**^*P*<0.01, ^***^*P*<0.001.

Table S7. Association of cigarette exposure with sex steroid hormone levels among males and females

| Cigarette exposure | TST | | EST | | SHBG | |
| --- | --- | --- | --- | --- | --- | --- |
|  | Male | Female | Male | Female | Male | Female |
| Smoking status |  |  |  |  |  |  |
| Never | 0 (ref.) | 0 (ref.) | 0 (ref.) | 0 (ref.) | 0 (ref.) | 0 (ref.) |
| Former | 0.000 (-0.037, 0.037) | 0.025 (-0.070, 0.131) | -0.034 (-0.071, 0.004) | 0.112 (-0.051, 0.302) | -0.032 (-0.071, 0.009) | 0.015 (-0.059, 0.094) |
| Current | 0.053 (0.006, 0.103) ^*§^ | 0.109 (0.019, 0.207) ^*^ | -0.031 (-0.070, 0.009) | -0.080 (-0.200, 0.059) ^§^ | 0.074 (0.027, 0.124) ^**§§^ | -0.005 (-0.080, 0.075) |
|  |  |  |  |  |  |  |
| Pack-years of smoking | 0.000 (-0.001, 0.001) | 0.000 (-0.003, 0.003) | -0.001 (-0.002, 0.001) | -0.001 (-0.005, 0.002) | 0.000 (-0.001, 0.001) | 0.001 (-0.002, 0.001) |
| Never-smokers | 0 (ref.) | 0 (ref.) | 0 (ref.) | 0 (ref.) | 0 (ref.) | 0 (ref.) |
| Q1 | 0.029 (-0.031, 0.093) | 0.040 (-0.036, 0.122) | 0.001 (-0.050, 0.055) | -0.061 (-0.233, 0.150) | 0.018 (-0.041, 0.080) | -0.019 (-0.114, 0.086) |
| Q2 | 0.023 (-0.043, 0.095) | 0.065 (-0.060, 0.207) | -0.051 (-0.092, -0.007) ^*^ | 0.127 (-0.085, 0.389) | -0.004 (-0.057, 0.052) | -0.032 (-0.103, 0.045) |
| Q3 | 0.037 (-0.019, 0.097) | 0.137 (-0.006, 0.301) | -0.049 (-0.082, -0.014) ^*^ | 0.026 (-0.143, 0.229) | 0.017 (-0.038, 0.076) | 0.039 (-0.065, 0.155) |
| Q4 | -0.002 (-0.066, 0.066) | 0.038 (-0.086, 0.180) | -0.045 (-0.110, 0.026) | -0.028 (-0.195, 0.173) | 0.026 (-0.036, 0.091) | 0.021 (-0.070, 0.120) |
| *P*_trend_ | 0.552 | 0.156 | 0.030 | 0.884 | 0.343 | 0.634 |
|  |  |  |  |  |  |  |
| Cigarettes per day now | 0.003 (0.0005, 0.006) ^*^ | 0.006 (-0.0001, 0.012) | -0.002 (-0.004, 0.001) | -0.006 (-0.014, 0.002) | 0.004 (0.002, 0.007) ^**^ | 0.000 (-0.004, 0.004) |
| Non-smokers | 0 (ref.) | 0 (ref.) | 0 (ref.) | 0 (ref.) | 0 (ref.) | 0 (ref.) |
| Q1 | 0.089 (-0.003, 0.190) | 0.018 (-0.080, 0.128) | 0.039 (-0.039, 0.122) | -0.280 (-0.467, -0.029) ^*^ | 0.053 (-0.029, 0.141) | 0.009 (-0.110, 0.144) |
| Q2 | 0.034 (-0.031, 0.103) | 0.143 (0.020, 0.279) ^*^ | -0.007 (-0.064, 0.053) | -0.008 (-0.175, 0.193) | 0.068 (0.007, 0.132) ^*^ | -0.074 (-0.175, 0.040) |
| Q3 | 0.024 (-0.077, 0.137) | 0.204 (0.052, 0.378) ^*^ | -0.040 (-0.119, 0.045) | -0.133 (-0.322, 0.109) | 0.126 (0.039, 0.219) ^**^ | 0.004 (-0.085, 0.102) |
| Q4 | 0.077 (0.012, 0.146) ^*^ | 0.052 (-0.072, 0.192) | -0.043 (-0.100, 0.017) | -0.061 (-0.225, 0.139) | 0.089 (0.014, 0.170) ^*^ | 0.012 (-0.070, 0.102) |
| *P*_trend_ | 0.028 | 0.032 | 0.139 | 0.211 | 0.001 | 0.893 |

Models were adjusted for age, BMI, race, educational qualification, physical activity, alcohol consumption, ratio of family income to poverty, regular periods, and batch (survey cycle). *P*_trend_ was tested by including never-/non-smokers, Q1, Q2, Q3, and Q4 as 1, 2, 3, 4, 5 (continuous variable) in models, respectively. All values were calculated using exp^β^-1, due to the natural logarithmic transformation of sex steroid hormones. ^*^*P*<0.05, ^**^*P*<0.01, ^***^*P*<0.001. ^§^former smokers were as a reference. ^§^*P*<0.05, ^§§^*P*<0.01, ^§§§^*P*<0.001.


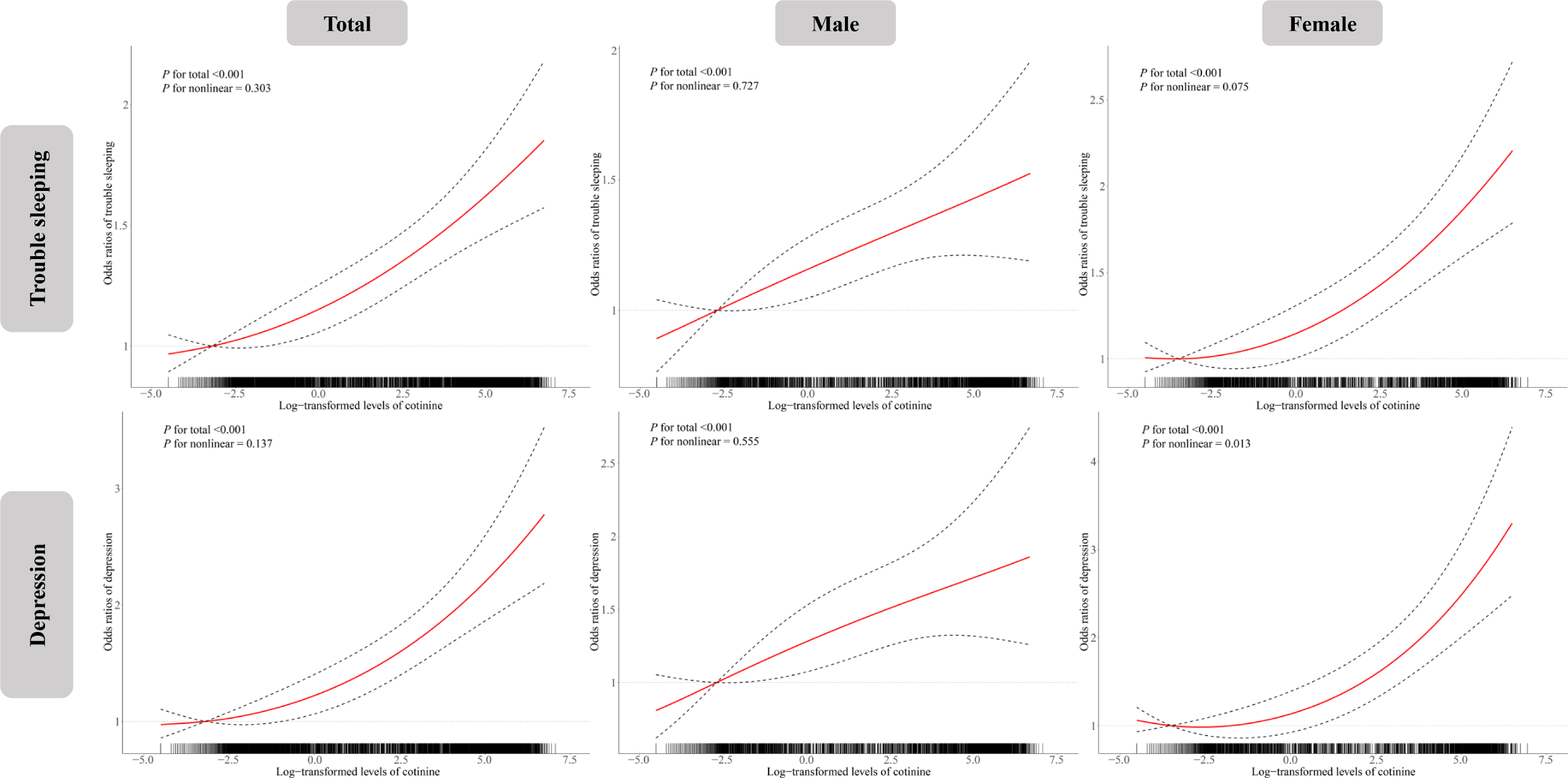


**Figure S2. Association of serum cotinine with trouble sleeping and depression.** Models were adjusted for age, gender, BMI, race, educational qualification, physical activity, alcohol consumption, ratio of family income to poverty, regular periods, and batch (survey cycle). Knots were placed at the 5th, 50th, and 95th percentiles of sex steroid hormone distributions, and the reference value was set at the 50th percentile.


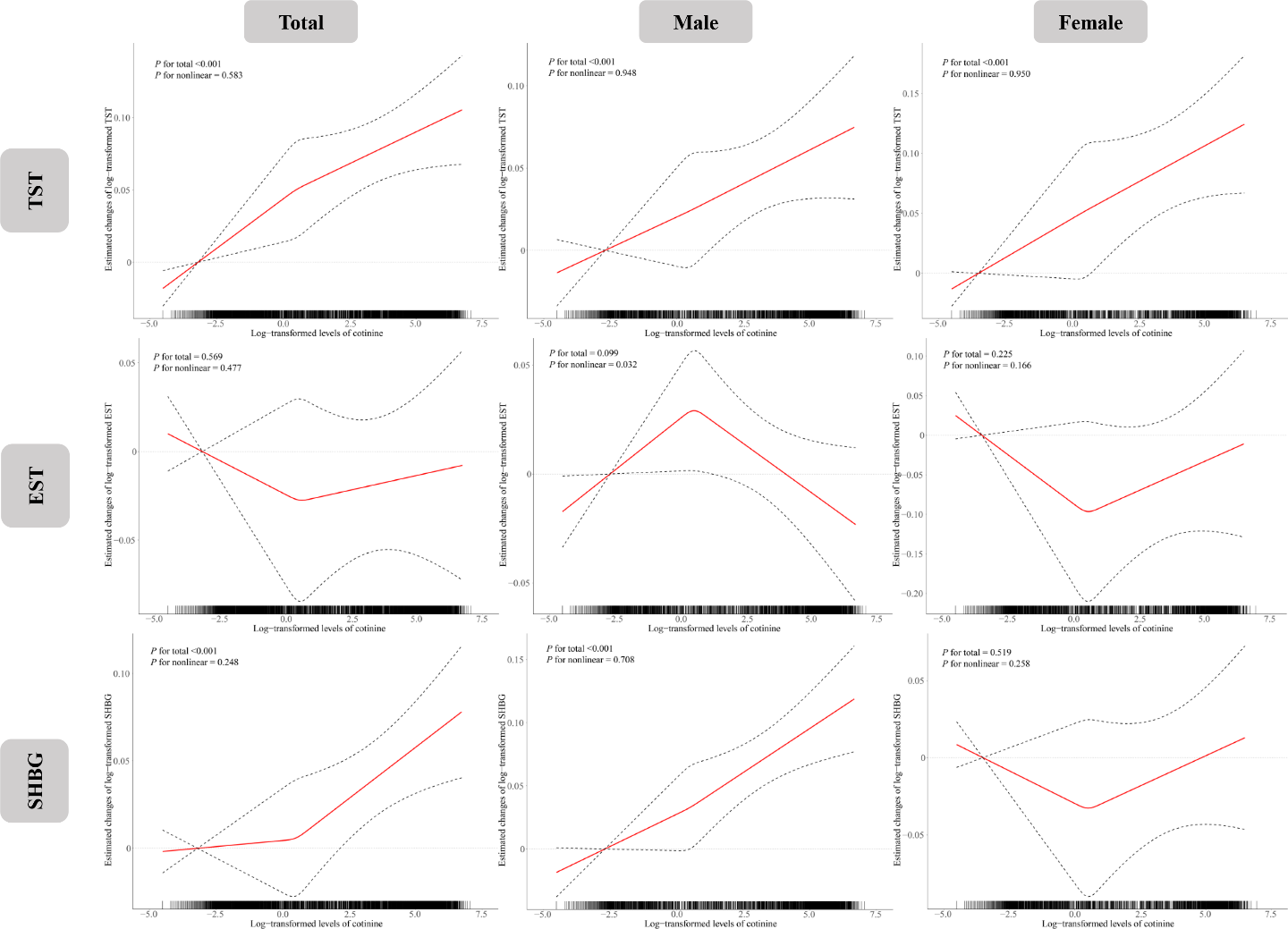


**Figure S3. Association of serum cotinine with sex steroid hormones.** Models were adjusted for age, gender, BMI, race, educational qualification, physical activity, alcohol consumption, ratio of family income to poverty, regular periods, and batch (survey cycle). Knots were placed at the 5th, 50th, and 95th percentiles of sex steroid hormone distributions, and the reference value was set at the 50th percentile.


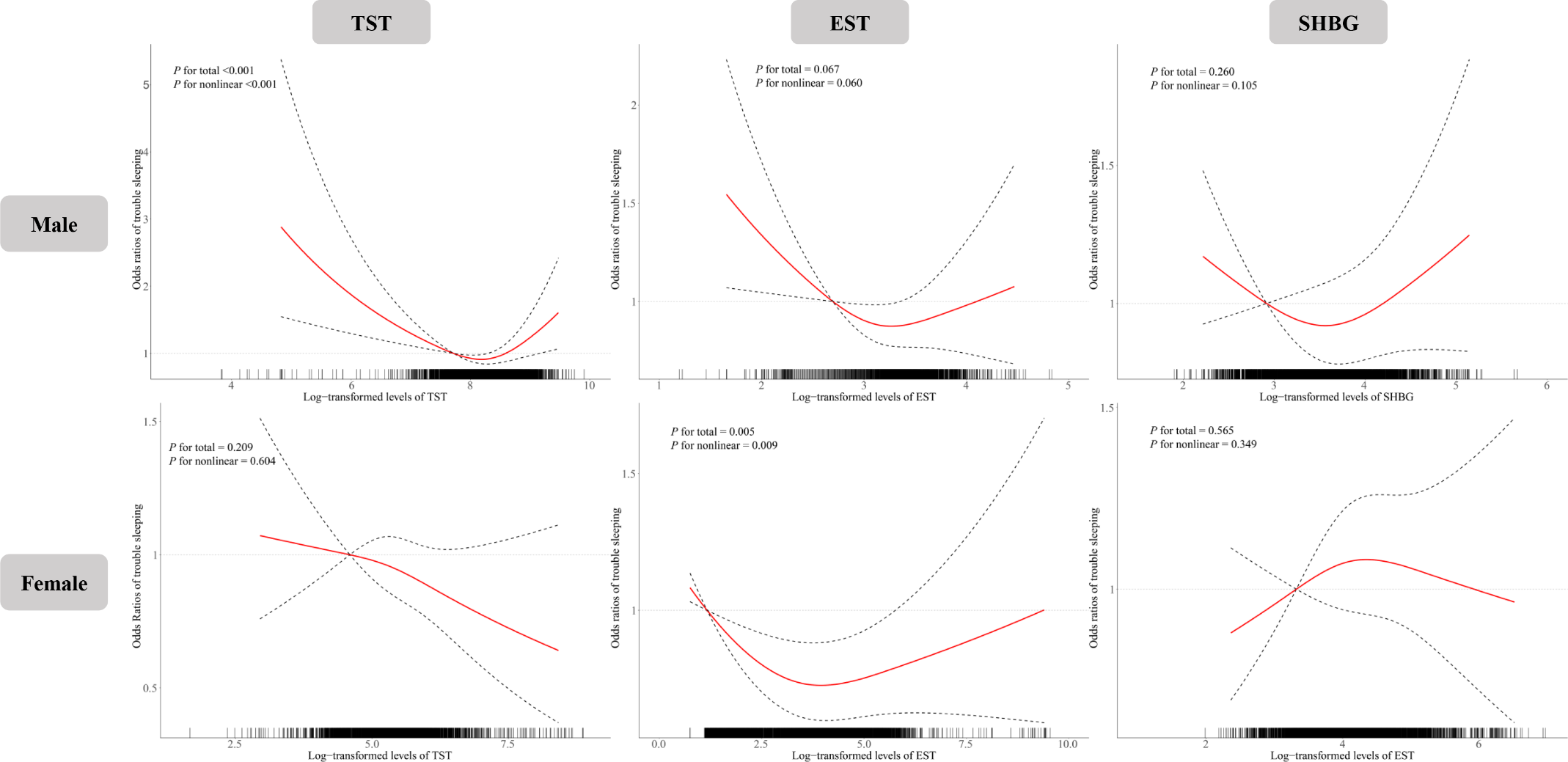


**Figure S4. Association of sex steroid hormones with trouble sleeping among males and females.** Models were adjusted for age, gender, BMI, race, educational qualification, physical activity, alcohol consumption, ratio of family income to poverty, regular periods, and batch (survey cycle). Knots were placed at the 5th, 50th, and 95th percentiles of sex steroid hormone distributions, and the reference value was set at the 10th percentile.


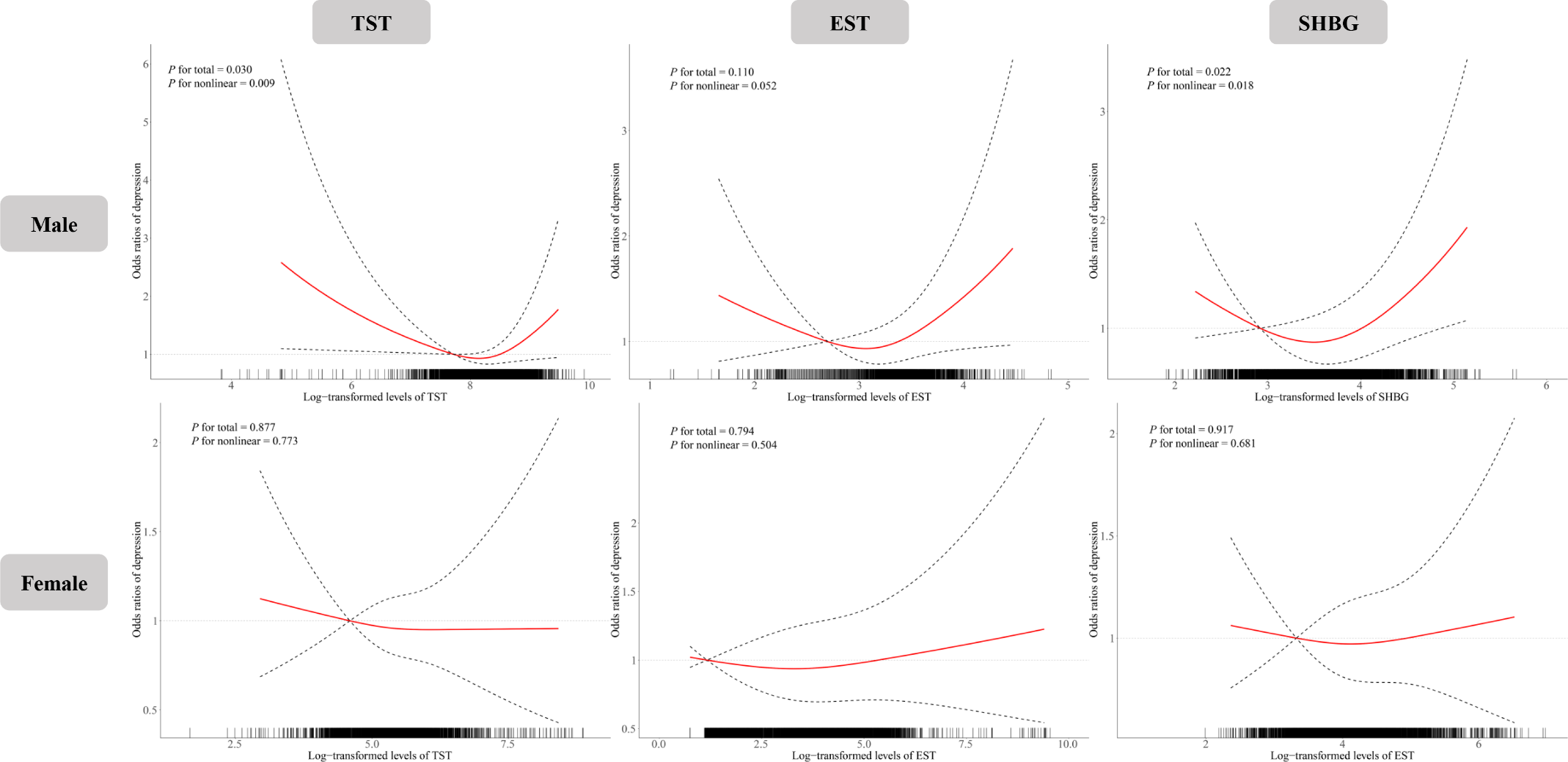


**Figure S5. Association of sex steroid hormones with depression among males and females.** Models were adjusted for age, gender, BMI, race, educational qualification, physical activity, alcohol consumption, ratio of family income to poverty, regular periods, and batch (survey cycle). Knots were placed at the 5th, 50th, and 95th percentiles of sex steroid hormone distributions, and the reference value was set at the 10th percentile.
